# Regulatory T Cells Diminish HIV Infection in Dendritic Cells – Conventional CD4^+^ T Cell Clusters

**DOI:** 10.3389/fimmu.2014.00199

**Published:** 2014-05-08

**Authors:** Maria E. Moreno-Fernandez, Jara J. Joedicke, Claire A. Chougnet

**Affiliations:** ^1^Division of Immunobiology, Department of Pediatrics, Cincinnati Children’s Hospital Research Foundation, Cincinnati, OH, USA; ^2^Immunology Graduate Program, College of Medicine, University of Cincinnati, Cincinnati, OH, USA; ^3^Institute for Virology, University Hospital Essen, University Duisburg-Essen, Essen, Germany

**Keywords:** regulatory T cells, HIV-1, dendritic cells, cyclic AMP, CTLA-4, actin cytoskeleton

## Abstract

Formation of immunological synapses (IS) between dendritic cells (DCs) and conventional CD4^+^ T cells (Tcon) is critical for productive immune responses. However, when DCs are HIV-infected such synapses are critical to establish HIV infection. As regulatory T cells (Treg) control DC–Tcon interactions, we inquired whether Treg might interfere with DC to Tcon HIV infection. We developed a model, using monocyte-derived DC infected with R5-HIV, and cultured with Tcon in the presence or absence of autologous Treg, using the physiological ratio of 1 Treg for 10 Tcon. Cultures containing Treg significantly decreased HIV infection in DC:T cell clusters. Notably, Treg appear to have an effect on the quality of the IS, as Treg decreased actin polymerization and DC maturation. Importantly, Treg decreased the trafficking of HIV punctate to the IS. Further, CD152 and cyclic adenosine monophosphate were critical Treg effector molecules, as their individual or simultaneous blockade abolished Treg activity, however no additive effect was found. Together, these data suggest that Treg can reduce HIV dissemination, which may be beneficial to the host in the early stages of infection.

## Introduction

Dendritic cells (DCs) constitute an important cellular targets for HIV. In particular, they are amongst the first target cells to encounter the virus at mucosal surfaces. DC can be directly and productively infected by HIV (1), and the newly generated HIV virions can infect other target cells through a process called *cis*-infection (2). Myeloid DCs are particularly sensitive to CCR5-using virus infection (3). Additionally, DCs can transmit virus to CD4^+^ T cells, without being themselves productively infected, through a process called *trans*-infection (4). HIV induces partial maturation of DC, which leads to their migration to the draining lymph nodes (5). Thus, DC harboring HIV mediates the spread of HIV to CD4^+^ T cells in a more efficient manner than cell-free virus (6). Similarly, DC:Tcon clusters are the main focus of infection *in vitro* (7). Consequently, the frequency and quality of DC–CD4^+^ T cell interactions plays a critical role in the efficiency of HIV transmission and spread.

Importantly, one of the mechanisms that can affect these interactions is regulatory T cells (Treg). Treg limit DC:T cell interactions by decreasing both DC maturation and T cell activation (8). Among the mechanisms used by Treg to suppress DC maturation, previous studies have implicated CD152 (CTLA-4) and cyclic adenosine monophosphate (cAMP) (9, 10).

We and others have reported a rapid increase in Treg frequency in all immune compartments of HIV-infected individuals (11, 12), which start during acute infection (13). We and others have recently shown that Treg can reduce the infection of conventional T cells and macrophages *in vitro* (14, 15). Interestingly, decreased infection of macrophages consequently attenuated HIV-associated neurodegeneration (14). However, the effect exerted by Treg on DC-mediated HIV infection of conventional CD4^+^ T cells (Tcon) has not yet been reported.

In the present study, we show that Treg significantly, albeit modestly, decreased HIV infection in DC:Tcon clusters. Our results also indicate that DC are the main targets of Treg, with a clear reduction of actin polymerization at the immunological synapse (IS), associated with a modest decrease of DC maturation. Treg decreased the mobilization of HIV punctate to the IS potentially due to their effect on actin polymerization. Mechanistically, individual or combined blockade of either cAMP or CTLA-4 was sufficient to abolish the Treg effect, however no additive effect was found.

## Materials and Methods

### Human subjects

Blood samples from healthy HIV-negative adult subjects, recruited by the Hoxworth Blood Bank (Cincinnati, OH, USA), were used in this study. Because the samples were not collected for research purpose and were de-identified, the University of Cincinnati Institutional Review Board had determined this activity to be exempted from IRB review and surveillance.

### Cell isolation and culture

Peripheral blood mononuclear cells (PBMCs) were separated by centrifugation over Ficoll-Hypaque (GE, Fairfield, CT, USA). CD14^+^ monocytes were isolated by positive selection (CD14 beads, Miltenyi Biotec, Auburn, CA, USA) and immature monocyte-derived DCs were generated by culturing the isolated monocytes for 4 days in complete medium (RPMI 1640, supplemented with 10% of heat-inactivated fetal calf-serum, HEPES, Glutamine) with 500 U/ml rhIL-4 and 1,000 U/ml rhGM-CSF (both from PeproTech, Inc., Rocky Hill, NJ, USA). Complete medium, including cytokines, was replaced at day 3. After 4 days of differentiation, cells had the morphology of immature DCs (CD14^low^ and HLA-DR^hi^ expression; no adherence to the culture plate). Resting autologous CD4^+^ T cells were purified by negative selection using the Miltenyi CD4 separation kit (Auburn, CA, USA), according to the manufacturer’s instructions. Purified CD4^+^ T cells were then stained with anti-CD8-FITC, anti-CD25-APC (BD Pharmingen; San Diego, CA, USA), and anti-CD127-PE (Beckman Coulter, Fullerton, CA, USA), to separate Treg and Tcon by cell sorting (FACSAria, BD). The purity of Treg (CD8^neg^CD25^hi^CD127^low^) and Tcon (CD8^neg^CD25^low^CD127^hi^) cells was evaluated post-sorting by CD4 and FOXP3 staining (clone PCH101, e-Bioscience; San Diego, CA, USA). Purity of the sorted populations was superior or equal to 90% (data not shown).

### Virus production and DC infection

The BaL HIV virus was obtained from the NIH AIDS Research and Reference Reagent Program. Simian Immunodeficiency Virus (SIV_mac_)-virion like-particles (VLPs) were obtained from Dr. Andrea Cimareli (Ecole Normale Supérieure, Lyon, France), as described (16). R5-tropic iGFP-JRFL HIV was obtained from Dr. Benjamin Chen (Mount Sinai Medical School, NY, USA), as described (17). 293 T cells were transfected with plasmids encoding either VLPs or complete iGFP-JRFL HIV, using the FuGENE transfection technology (Roche) according to the manufacturer’s protocol. After 2 days, supernatants were harvested, and viruses were concentrated using 100 K filtration systems (Millipore). BaL and iGFP-JRFL were titrated using the TZM-bl indicator cell line. VLP titers were determined using the Reverse Transcriptase colorimetric assay kit (Roche). Immature DCs were infected at a multiplicity of infection (MOI) of two. Briefly, DCs were incubated with HIV (BaL or iGFP-JRFL) plus SIVMAC-VLPs for 2 h at 37°C, plated at 0.5 × 10^6^ per well in 1 ml of complete media and cultured for 3 days to allow for productive infection. In experiments with iGFP-JRFL viruses, cell sorting was used to enrich infected DCs, based on their GFP expression (FACSAria, BD). In some experiments, DCs were treated with Zidovudine (AZT, 1 μM) at the time of HIV infection to inhibit productive infection.

### Co-culture of DC, Tcon, and Treg

After 3 days of infection, DCs were washed twice with complete RPMI and co-cultured for 24 h with autologous Tcon stained with 0.3 μM carboxyfluorescein diacetate succinimidyl ester (CFSE; Molecular Probes; Eugene, OR, USA), in the presence or absence of autologous Treg stained with Calcein AM Blue (Molecular Probes). Ratios of 1 DC, 10 Tcon, and 1 Treg were used. For the experiments using DC infected with iGFP-JRFL, Tcon were stained with Cell trace Violet (Molecular Probes). Co-cultures were carried out for 36 h, to evaluate the effect of Treg on viral trafficking from DC to Tcon. This time was chosen after kinetics studies, because the virus was found in DC, IS, and Tcon at that time, while the numbers of DC:T cell clusters remained high.

### Blockade of Treg activity

In some experiments, co-cultures were performed in presence of blocking anti-human CD152 (CTLA-4, 10 μg/ml, clone BNI3, Beckman Coulter). In other experiments, Treg were pre-treated for 24 h with 2′,5′-Dideoxyadenosine (ddADA), an inhibitor of adenyl cyclase (200 μM, Sigma). Treg were then extensively washed to avoid a direct contact of ddADA with the DC and Tcon. In addition, simultaneous blockade of both pathways was performed in some experiment.

### Flow cytometry analysis of HIV-p24^Gag^ levels, DC maturation, and T cell activation markers

Cells in co-cultures were harvested, treated with 20 μg/ml of human IgG to block Fc receptors, and stained with anti-HLA-DR eFluor450 (Clone L243; eBiosciences, San Diego, CA, USA), anti-CD80 AF-647 (Clone MEM-233, AbD Serotec, Raleigh, NC, USA), anti-CD83 PE-Cy7 (Clone HB15e, eBiosciences), anti-CD86 AF-647 [Clone 2331 (FUN-1), BD; San Diego, CA, USA], anti CD40-APC-H7 (Clone 5C3, BD), anti-CD25 AF700 (Clone M-A251, BD), anti-CD69 PE-Cy7 (Clone FN50, Biolegend, San Diego, CA, USA), anti-CD134 PerCP-Cy5.5 (Clone ACT35, Biolegend), and Live/Dead^®^ Fixable Aqua Dead Cell Stain Kit (Invitrogen) for 30 min at room temperature in PBS containing 2% fetal calf-serum and 0.1% sodium azide. Cells were then washed, fixed with 2% formaldehyde for 30 min at 4°C, and stained with anti-HIV-p24^Gag^ PE (Clone KC-57; Beckman Coulter), in 0.3% saponin buffer for 30 min at 4°C. A minimum of 100,000 events were acquired for each sample and analyzed on a LSRII (BD), using the DIVA software. Live cells were gated based on forward- and side-scatter properties and the absence of fluorescence in the Live/Dead Viability Assay. Tcon were identified based on CFSE expression, DCs were gated on the expression of HLA-DR, and clusters were gated based on forward-scatter and double expression of CFSE and HLA-DR.

### Determination of Tcon:DC clusters and actin polymerization at the IS by imaging flow cytometry

DC:Tcon:Treg co-cultures were harvested, treated with 20 μg/ml of human IgG, and stained with anti-HLA-DR PerCP (Clone L243, BD) for 30 min at 4°C. Cells were then fixed with 2% methanol-free formaldehyde for 30 min, washed, and stained with Phalloidin AF-647 in 0.3% saponin for 30 min at 4°C. At least 50,000 events/sample were acquired on the ImageStream (Amnis, EMD Millipore) and analyzed using the IDEAS Software version 5.0. To evaluate the number of Tcon interacting with each DC in each cluster, the number of CFSE^+^ cells per HLADR^+^ cell was quantified using the Spot count feature. To quantify the actin polymerization at the IS, we used the interface mask. We calculated the intensity of actin within the interface, as well as that in the overall DC cluster. In each sample, we analyzed only the clusters in which it was possible to position a IS mask without ambiguity (~100 events/sample). Data were normalized based on the total actin intensity in the overall cluster. Data are calculated with the following formula (actin intensity at the interface mask/total actin). As control for actin polymerization, cells were treated with Cytochalasin D (20 μM, Sigma) at the time of co-culture.

### Determination of iGFP-HIV viral trafficking by imaging flow cytometry

Co-cultures were harvested 36 h after initiation, treated with 20 μg/ml of human IgG and stained with anti-HLA-DR BV570 (Clone L243, Biolegend) for 30 min at 4°C. Cells were then fixed with 2% methanol-free formaldehyde for 30 min, washed, and stained with phalloidin AF-647 in 0.3% saponin for 30 min at 4°C. At least 50,000 events were acquired for each sample and analyzed using the IDEAS Software version 5.0. To evaluate the HIV migration to the IS, we first quantified the iGFP-HIV intensity at the synapse divided by the total iGFP-HIV intensity. Second, we quantified the actin intensity at the interface divided by the total actin staining. Third, we plotted these two features to track the co-localization of HIV with polymerized actin at the IS. In some experiments, DCs were treated with Zidovudine (AZT, 1 μM) at the time of HIV infection to confirm HIV infection. In addition, cells were treated with Cytochalasin D (20 μM) or Nocodazole (100 μM) at the time of co-culture, as control of actin polymerization and viral movement.

### Cytokine quantification in culture supernatants

IL-2, IL-6, IL-10, IL-12p40, TNF-α, and IFN-γ levels were quantified in the 24 h co-culture supernatants using Luminex assays (Millipore, Billerica, MA, USA).

### Statistical analysis

Statistical analyses were performed using Prism (GraphPad Software 5). Paired *t*-tests were used to compare different culture conditions. A *p* value of ≤0.05 was considered to be significant. Correlations coefficient and *r* were calculated using Pearson correlation. To compare the correlation coefficients, we used the method reported by http://core.ecu.edu/psyc/wuenschk/docs30/CompareCorrCoeff.pdf.

## Results

### Treg decrease the frequency of DC:Tcon clusters with evidence of productive HIV infection

**Figure 1:**
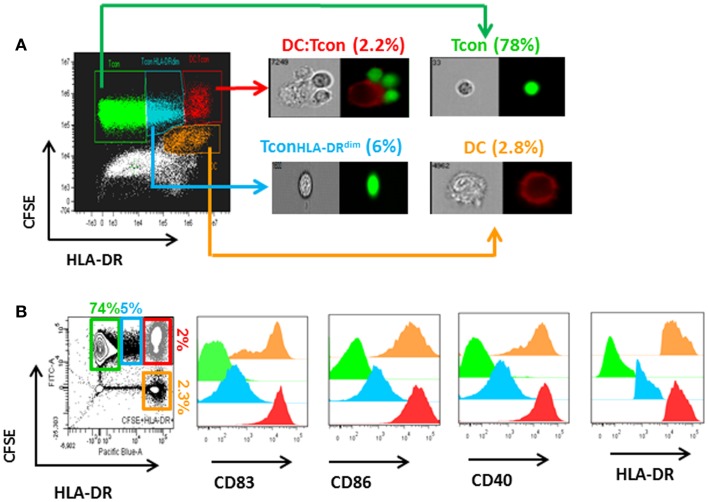
**Analysis of DC:Tcon clusters using a combined imaging and classic flow cytometry strategy**. DC were infected with HIV_BaL_ for 3 days and cultured with CFSE-labeled Tcon for 24 h at a 1:10 ratio. **(A)** Using imaging flow cytometry ImageStream), DC:Tcon clusters were identified by the expression of CFSE and HLA-DR. Isolated Tcon were CFSE^+^HLADR^−^ (green gate), isolated activated Tcon were CFSE^+^HLADR^dim^ (blue gate), DC:Tcon were CFSE^+^HLADR^+^ (red gate), and isolated DC are CFSE-HLADR^+^ (orange gate) populations. DC:Tcon clusters were found only in the CFSE^+^LADR^+^ population. **(B)** Flow cytometry analysis of CD83, CD86, CD40, and HLA-DR expression in isolated Tcon (CFSE^+^HLADR^−^; green histograms), isolated semi activated Tcon (CFSE^+^HLADR^dim^; blue histograms), DC:Tcon (CFSE^+^HLADR^+^; red histograms), and isolated DC (CFSE^−^HLADR^+^; orange histograms) populations.

To test the hypothesis that Treg can control HIV infection of DC:Tcon clusters, we used an *in vitro* model of *cis*-infection where HIV-infected DCs were cultured with autologous Tcon in the presence of anti-CD3 to induce IS formation. Importantly, the Treg:Tcon ratio we used (1:10) is similar to the proportion found in lymphoid tissues. Tcon were labeled with CFSE before the co-culture and, after 24 h of co-culture, HLA-DR expression was used to identify the DC. Using imaging flow cytometry, DC:Tcon clusters were identified by their dual expression of CFSE and HLA-DR. The isolated Tcon and the DCs were identified by the single expression of CFSE or HLA-DR, respectively. Morphology of the cells confirmed that this analysis strategy was correct (Figure 1A). Using the same gating strategy in classic flow cytometry led to similar frequency of CFSE^+^HLA-DR^+^ clusters (Figure 1B, left panel). Additionally, we looked at several DC markers, and, as shown in Figure 1B, DC:Tcon clusters (red histograms) highly expressed CD83, CD86, and CD40, at levels comparable to those of DC alone (orange histograms), while isolated Tcon (CFSE^+^HLA-DR^−^, green histograms) did not express these molecules. Some CFSE^+^ cells were expressing low levels of HLA-DR, and could be activated Tcon. Confirming that it was the case, expression of CD83, CD86, and CD40 by these CFSE^+^HLA-DR^low^ cells was lower than those observed in the DC:Tcon clusters (blue histograms, Figure 1B). They also had a typical T cell morphology (Figure 1A). Together, these analyses established that the gating based on CFSE and HLA-DR^hi^ expression consistently identified DC:Tcon clusters, and not activated Tcon or Tcon:Tcon clusters.

**Figure 2:**
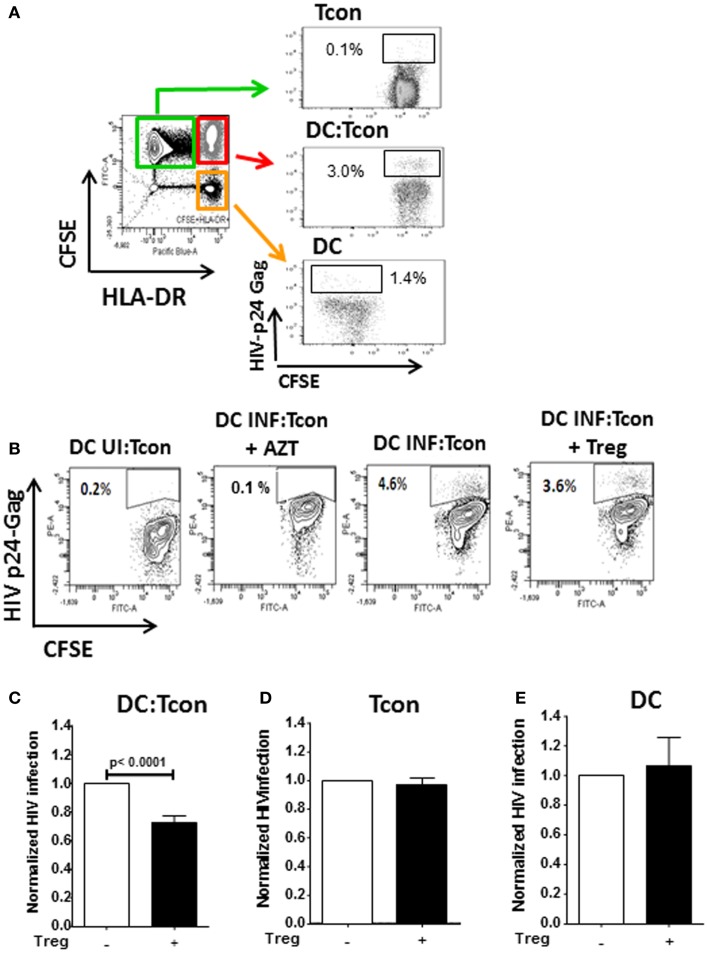
**Infection of DC:Tcon clusters is decreased by Treg, but infection of isolated Tcon or DC remains unchanged**. DCs were infected with HIV_BaL_ for 3 days and cultured with CFSE-labeled Tcon for 24 h at a 1:10 ratio. **(A)** After exclusion of dead cells, the percentage of HIV-p24Gag^+^ cells was measured in the gated Tcon (CFSE^+^HLADR^−/dim^), DC:Tcon (CFSE^+^HLADR^+^), and DC (CFSE^−^HLADR^+^) populations. **(B)** Treg were added or not to DC:Tcon cultures at a 1:10:1 (DC:Tcon:Treg) ratio and co-cultured for 24 h. After exclusion of dead cells, the percentage of HIV-p24Gag^+^ in clusters, single Tcon, or single DC was analyzed by flow cytometry using the gating strategy described in **(B)**. Cultures containing AZT were used to set up the HIV-p24Gag^+^ gate. One representative experiment is shown. Percentage of HIV-p24Gag^+^ cells is indicated in each panel. UI, uninfected; INF, infected. **(C–E)** Summary of all experiments (*n* = 12). **(C)** DC:Tcon clusters, **(D)** single Tcon, and **(E)** single DC. Normalized HIV infection was calculated based on the DC:Tcon cultures without Treg for each individual. Mean (SE) percent infection in DC:Tcon clusters, Tcon, and DC are shown. *p* Values correspond to paired *t*-tests.

As shown in Figure 2A, HIV infection mainly occurred in the DC:Tcon (CFSE^+^HLADR^+^) clusters (mean percentage of infection: 3.9 ± 0.5%), compared to either isolated Tcon (CFSE^+^HLADR^−^, mean percentage of infection: 0.8 ± 0.3%), DC (CFSE^−^HLADR^+^, mean percentage of infection: 2.4 ± 0.6%), or the CFSE^−^HLADR^−^ population (mean percentage of infection: 0.9 ± 0.2%). As expected (18), there was a significant variability among donors and the infection in the clusters ranged from 1.6 to 8%. Addition of Treg significantly decreased the percentage of infected DC:Tcon clusters (*p* = 0.001, Figure 2B shows a representative experiment and Figure 2C displays the summary of 12 independent experiments). This decrease was seen in all individuals we studied. In contrast, infection level of isolated Tcon or DC was not changed by Treg (*p* = 0.36 and *p* = 0.38, respectively, Figures 2D,E), suggesting that DC:Tcon clusters are the main targets of Treg suppression at this early time point. Of note, the effect of Treg on HIV infection was not due to enhancement of HIV infection by co-infection with VLP, because Treg also decreased infection in DC:clusters when DCs were infected with BaL alone (Figure S1 in Supplementary Material). Treg could also form aggregates with DC. Infection frequency in these aggregates was 3.4 ± 2.8%, which was similar to that of DC:Tcon clusters (*p* = 0.45). Treg infection might account for decreased Tcon infection, due to possible competition for access to infected DCs. Such mechanism would be suggested by an inverse correlation between frequency of infected DC:Treg clusters and that of infected DC:Tcon aggregates. However, no such correlation existed (*r* = 0.34 and *p* = 0.19).

### Treg do not alter DC:Tcon cluster formation or Tcon activation but modestly decreased DC maturation

**Figure 3:**
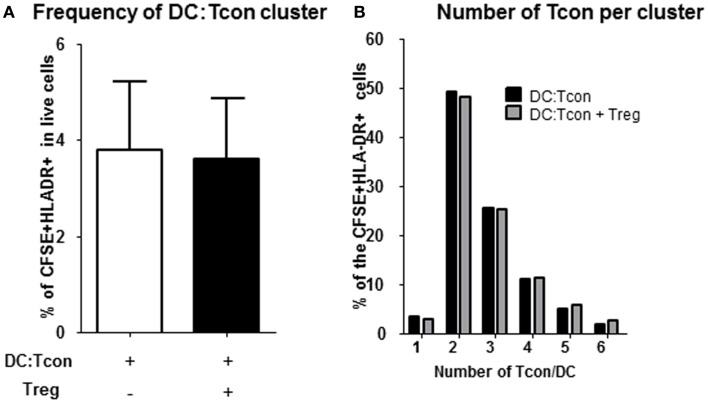
**Regulatory T cells do not decrease formation of DC:Tcon clusters nor their composition**. **(A)** Cluster frequency in the absence and presence of Treg was quantified by flow cytometry (*n* = 6). *p* Value corresponds to a paired *t*-test. **(B)** Number of Tcon in each DC:Tcon cluster was quantified by imaging flow cytometry, in the absence and presence of Treg using a spot count analysis (*n* = 925–1,115/condition). One representative experiment of 2 is shown.

Regulatory T cells have previously been shown to impair T cell activation by decreasing the interaction and contact time between CD4^+^ T cells and DC when high Treg:Tcon ratios (1:1) were used (8, 19). At the more physiological frequency we used (1:10 Treg:Tcon), Treg did not impair the formation of DC:Tcon clusters (Figure 3A). Next, we evaluated by flow cytometry whether Treg changed the composition of DC:Tcon clusters. Most DC interacted with 2 Tcon (Figure 3B) and the presence of Treg did not affect the number of Tcon that interacted with each DC.

Regulatory T cells can decrease CD4 activation and DC maturation (8, 20), which could explain decreased HIV infection in the presence of Treg. Although Treg significantly decreased the levels of expression of CD40 and CD83 by DC (both *p* = 0.01), they did not diminish their expression of CD80 and CD86 (Table 1). It must be noted that this effect was modest as the DC still expressed high levels of all the co-stimulatory molecules in presence of Treg. Treg did not affect the expression of CD25 and CD134 by Tcon (Table 1). Additionally, Treg did not alter the levels of IL-6, IL-2p40, IL-10, IL-2, TNF-α, and IFN-γ in the DC:Tcon culture supernatants (data not shown). Importantly, the effect of Treg on DC maturation was not due to the fact that DCs were infected with both BaL and VLP, because Treg also decreased DC maturation when DCs were infected with BaL alone. Indeed, decrease in CD40 MFI due to the presence of Treg (mean delta CD40 MFI) was 4,390 ± 1,193 in absence of VLP, compared to 4,061 ± 1,210 in their presence. Treg similarly decreased CD83 MFI in the presence or absence of VLP (mean delta CD83 MFI was 3,029 ± 2,818 in absence of VLP, compared to 2,173 ± 3,603 in their presence).

**Table 1:** **DC and Tcon phenotype**.

	DC INF:Tcon	DC INF:Tcon^+^Treg	*p* Value^#^
**DC PHENOTYPE**			
CD80^a^	3,799 ± 1,152	4,186 ± 1,226	0.07
CD83	15,193 ± 1,991	12,287 ± 2,041	0.01
CD86	64,219 ± 12,354	58,695 ± 9,427	0.81
CD40	37,257 ± 3,403	33,446 ± 3,306	0.01
**Tcon PHENOTYPE**			
CD25	4,450 ± 958	3,960 ± 864	0.25
CD134	6,153 ± 1,441	6,071 ± 1,588	0.46

*^a^ Values are expressed as mean MFI ± SEM (*n* = 10)*.

*^#^*p* Values correspond to paired *t*-test*.

### Actin polymerization at the IS is significantly decreased in presence of Treg

**Figure 4:**
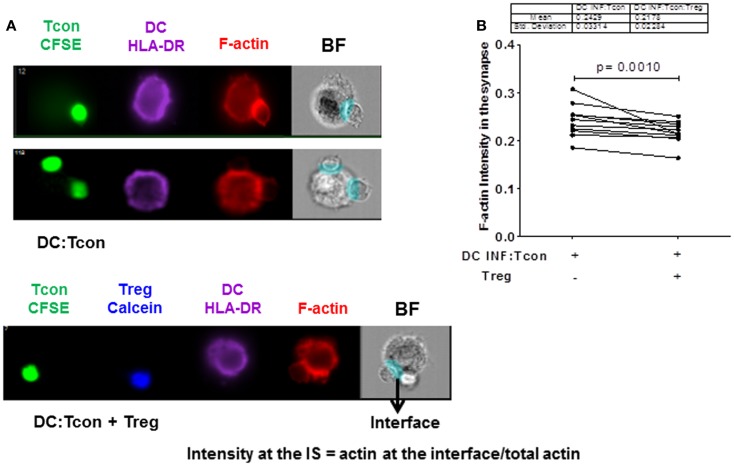
**Regulatory T cells decrease actin polymerization at the immunological synapse in DC:Tcon clusters**. The actin polymerization at the IS was quantified using interface mask. Intensity of actin within the interface was calculated, as well as that in the overall DC cluster. Data are expressed as a ratio of actin in the IS divided by the total actin intensity in the cluster. **(A)** The upper panel shows actin polymerization at IS in one DC:1 Tcon and one DC:2 Tcon cluster middle panel, the lower panel shows actin polymerization in the DC:Tcon IS in a DC:Tcon:Treg cluster. **(B)** Summary of 11 independent experiments, comparing actin polymerization in the absence and presence of Treg. *p* Value corresponds to a paired *t*-test.

We next investigated whether Treg interfered with the formation of a stable IS, because it constitutes a critical step for viral dissemination to T cells. In particular, actin polarization at the IS plays an important role in cell-to-cell viral spread (21). We visualized actin polymerization in the DC:Tcon clusters by imaging flow cytometry. Cytochalasin D treatment abolished actin polymerization at the IS, which confirms the validity of our analysis (Figure S2 in Supplementary Material). As shown in Figure 4A (lower panel), we quantified polymerized actin at the IS. Treg significantly, albeit modestly, decreased IS actin polymerization (Figure 4B). The effect of Treg on actin polymerization at the IS was not due to the presence of VLP, because Treg similarly decreased actin polymerization at the IS when DCs were infected with BaL alone (not shown). Treg effect on actin polymerization was not due to the fact that more cells where bound to the DC, because when we restricted our analysis to aggregates that contain only one Tcon per DC, Treg also significantly decreased actin polymerization (*p* = 0.01).

### HIV punctate localization in DC:Tcon clusters is altered by the presence of Treg

**Figure 5:**
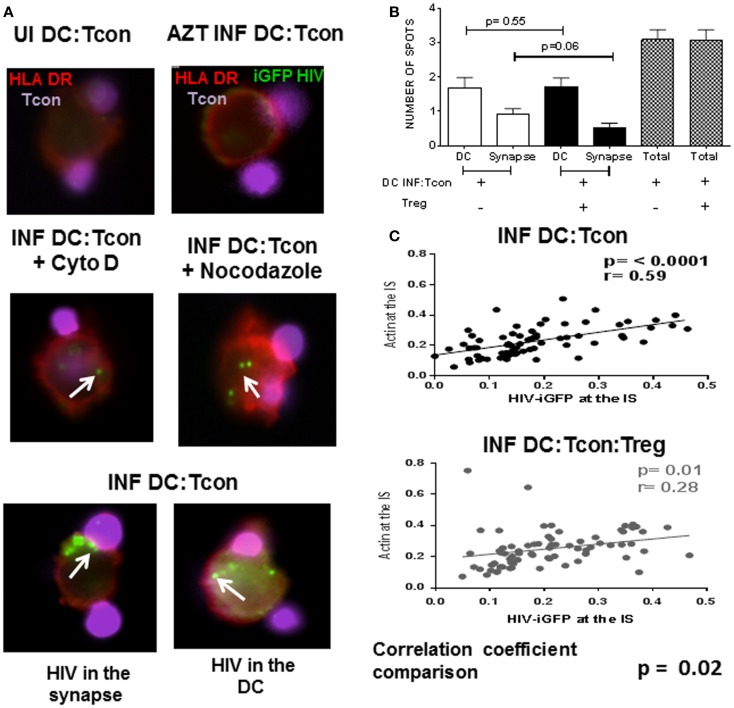
**HIV localization in DC:Tcon clusters is altered by the presence of Treg**. DCs were infected with HIV JRFL iGFP-expressing and enriched by cell sorting. DC:Tcon:Treg were co-cultured for 24 h in the presence or not of AZT (1 μM) or Cytochalasin D (20 μM) or Nocodazole (100 μM). **(A)** Upper panel shows the uninfected cells (UI) and the AZT-treated cultures. Tcon were labeled with Cell Violet Tracer (violet) and DCs were stained for HLA-DR (red). Middle panel shows the effect of Cytochalasin D and Nocodazole on HIV localization (indicated by white arrows). Lower panel shows HIV-iGFP in DC:Tcon clusters localized either at the IS (left) or inside the DC (right). **(B)** Summary of three independent experiments, comparing number of punctate at the IS or DC in the absence and presence of Treg. *p* Value corresponds to a paired *t*-test. **(C)** HIV migration to the IS was assessed first by quantifying the iGFP-HIV intensity at the synapse divided by the total iGFP-HIV intensity. Second, we quantified the actin intensity at the interface divided by the total actin staining as described in Figure 4. Third, we plotted these two parameters to track the co-localization of HIV with polymerized actin at the IS. Correlation of actin polymerization and HIV-iGFP localization in DC:Tcon clusters in the absence and presence of Treg. Correlation coefficients of the two data sets were compared (*p* = 0.02).

We next investigated whether Treg affected the mobilization of HIV toward the IS. We used the GFP-expressing CCR5 virus JRFL to infect DC and evaluated viral distribution in the GFP^+^ DC:Tcon clusters. HIV staining has a punctate pattern as previously described (17), which allowed quantification of its cellular localization in the clusters (Figure 5A). Treatment of DC with AZT before infection confirmed that this punctate staining was newly generated HIV viruses (Figure 5A, right upper panel). To validate our experimental system, co-cultures were treated with Cytochalasin D and Nocodazole, which inhibit actin and microtubule polymerization, respectively. As shown in Figure 5A (middle panels), Cytochalasin D and Nocodazole both inhibited HIV movement to the IS as reported (22). When we analyzed HIV localization, we found HIV mainly in DC and at the IS (Figure 5A lower panels). About 50% of the punctate was located in the DC, followed by around 25% at the IS, and only 20% of the virus was in the Tcon (data not shown). Notably, the presence of Treg decreased the number of punctate present at the IS, but did not change the total number of punctate present in the aggregates (Figure 5B), suggesting that Treg mainly alter viral distribution. Particularly, actin polymerization and HIV-iGFP localization at the IS were positively correlated in the DC:Tcon clusters (*p* ≤ 0.0001, Figure 5B upper panel). When Treg were present in the culture, this correlation still existed (*r* = 0.28, *p* = 0.01, Figure 5C lower panel), but the strength of association was lower. We formally tested whether Treg affected this localization by comparing the correlation coefficient of these two sets of data (method developed by R. A. Fisher) and found a significant difference (*p* = 0.02), suggesting that Treg decreased viral movement toward the IS.

### Treg-mediated decrease of viral infection in DC:Tcon clusters is mediated by their CTLA-4 or cAMP activity

**Figure 6:**
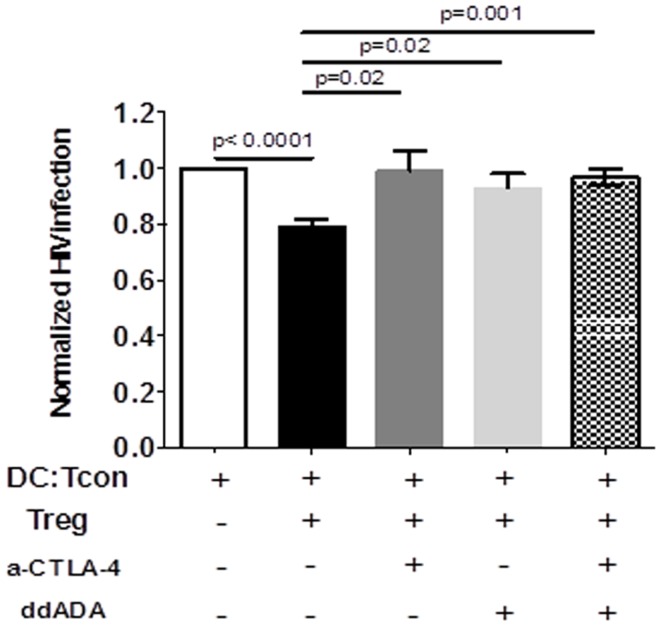
**Regulatory T cells-mediated suppression of viral infection is mediated by their CTLA-4 or cAMP activity**. DC:Tcon:Treg were co-cultured for 24 h in the presence or not of anti-CTLA-4 blocking antibody. Treg previously treated with an inhibitor of adenyl cyclase (ddADA) were added or not to DC:Tcon co-cultures. Percentage of HIV-infected Tcon in clusters was normalized based on corresponding cultures without Treg. Effect of individual CTLA-4 or cAMP and combined CTLA-4/cAMP blockade are shown. Mean and SE are shown (*n* = 12).

CTLA-4 and cAMP are mechanisms used by Treg to control DC interactions with Tcon or CD8^+^ T cells (23, 24). We therefore tested the involvement of these mechanisms in Treg activity by blocking simultaneously these two effector molecules. To assure that the blockade principally affected Treg function, we verified that the anti-CTLA Ab did not affect HIV infection in the clusters in absence of Treg (data not shown). Similarly, Treg but not DC or Tcon, were in contact with ddADA, because ddADA-treated Treg were extensively washed before co-culture. Importantly, blockade of both CTLA-4 and cAMP resulted in significant inhibition of Treg capacity to suppress HIV infection (Figure 6). Individual blockade of either cAMP or CTLA-4 was sufficient to abolish the Treg effect (Figure 6); however no additive effect was found.

### Decreased actin polymerization and DC maturation by Treg are the mechanism most associated with their effect on HIV replication

**Figure 7:**
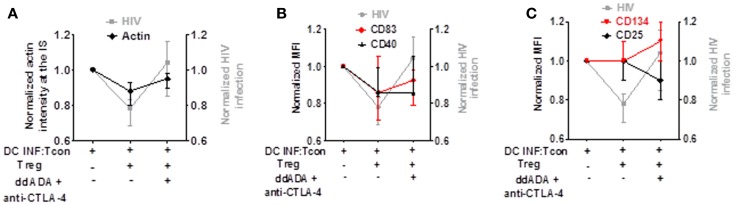
**Decreased actin polymerization and DC maturation by Treg is the effect most associated with their control of HIV replication**. Comparison of Treg effect and combined CTLA-4/cAMP blockage on HIV replication, actin polymerization, DC maturation, and Tcon activation. **(A)** Blockade of Treg function on HIV infection or actin polymerization. **(B)** Blockade of Treg function on HIV infection or CD83 and CD40 expression. **(C)** Blockade of Treg function on HIV infection or CD25 and CD134 expression. Median of 6 (for actin polymerization) and 7 (for CD83, CD40, CD25, and CD134) experiment is shown.

To determine which mechanism is contributing to Treg-mediated decrease of viral infection, we analyzed in parallel the effect of combined CTLA-4 and cAMP blockade on HIV replication, actin polymerization in the IS, DC maturation, and Tcon activation. As shown in Figures 7A–C, while the presence of Treg decreased HIV infection, actin polymerization at the IS, and DC maturation, it did not block Tcon activation. In addition, blockade of CTLA-4 and cAMP reversed Treg effect on HIV infection, actin polymerization in the IS (Figure 7A), and CD83 expression (Figure 7B). Together, these data thus suggest that the effect of Treg on cytoskeleton rearrangement and DC maturation are the most strongly associated with their effect on HIV infection.

## Discussion

Understanding the immunological mechanisms that underlie HIV progression remains of essential importance. The earliest interactions of the virus with its cellular targets are of particular relevance because these events will affect subsequent set points. DCs are amongst the first target cells to encounter the virus at mucosal surfaces (25, 26). It is also well established that DC facilitate HIV dissemination to the lymphoid organs by enabling HIV infection of CD4^+^ T cells (27, 28). Therefore, studying the mechanisms that control DC-mediated viral transmission is critical to our understanding of HIV infection. Although the biological effect of Treg during HIV infection remains uncertain, Treg have been shown to accumulate during HIV infection (3, 11, 13, 29) and to alter DC–T cells interactions [reviewed in Ref. (30)]. This Treg accrual could thus control DC-mediated transmission of HIV to CD4^+^ T cells, similar to their effect on HIV infection in macrophages and CD4^+^ conventional T cells (14, 15).

To address this question, we developed an *in vitro* model in which we could study how Treg affect viral infection of DC:Tcon clusters. An important characteristic of our model is that we used a ratio of 1:10:1 DC:Tcon:Treg, which mimics the frequency encountered in tissues (19). We used a R5 strain to infect the DC, as R5 viruses are the ones predominantly found in HIV^+^ patients (31, 32), and we focused our analysis on DC:Tcon clusters, because they are the primary site of DC-mediated HIV infection. Of note, we used a *cis*-infection model, in which DC were infected and kept in culture for 3 days before co-culture with Tcon and Treg. Previous studies have shown that, in this setting, viral particles that are endocyted and not integrated are rapidly degraded (33, 34). Thus, it is likely that the viruses detected in our experiments were newly assembling viral particles at the DC:Tcon IS and not endocytosed viruses that had been previously assembled, although we cannot formally exclude the later possibility.

In this model, Treg decreased the frequency of infected DC:Tcon clusters. Although the effect was modest, it was reproducible in all subjects. This effect was not due to a decreased frequency of DC:Tcon clusters nor was it due to Treg displacing Tcon from the clusters. Although a prior study reported such displacement (8), in our hands, Treg did not appear to strictly disrupt DC:Tcon conjugates. A potential explanation for this difference is the fact that a higher Tcon:Treg ratio (1:1) was used in the previous study, while we used the more physiological ratio of 10:1. In contrast, our results are consistent with another report showing that Treg did not decrease the capacity of DC and Tcon to form clusters, but they impaired the activation of T cells and their capacity to produce cytokines (35). In our model, Treg also partially decreased DC maturation; Treg diminished CD40 and CD83 levels while cytokine production and expression of CD80 and CD86 were not affected. Furthermore, T cell activation was not significantly changed by Treg. These data are in agreement with a previous study showing that Treg modestly reduce the expression of co-stimulatory molecules by DC, but it nevertheless impacted their ability to activate naïve CD4 T cells (20).

Regulatory T cells also reproducibly impacted actin polymerization and mobility of viruses toward the IS, although, as for DC activation, their effect was modest. However, even a modest disruption of HIV transfer due to actin cytoskeleton disruption may have impact HIV infection because cell-mediated viral transmission is such a critical step in the spread of HIV infection. Indeed, previous studies have shown that HIV-1 associates with actin during its intracellular maturation and assembly, thus favoring virion budding to the sites where DC and T cells are in contact (36, 37). HIV motility in infected cells is blocked by actin filament disassembly following latrunculin treatment (38). In the same line, disruption of actin or microtubule networks in DC and CD4^+^ T cells by specific cytoskeleton inhibitors inhibited DC–T cell interactions and cell-to-cell HIV spread (22). Interestingly, inhibition of actin cytoskeleton assembly by Cdc42 blockade did not prevent the formation of DC:T cell clusters, but decreased the transmission of HIV-1 to CD4 T lymphocytes (39). Additionally, blockage of Rho GTPase activation, and consequent inefficient actin cytoskeleton rearrangement, reduced HIV envelope fusion with target cells (40).

Our data suggest that diminished DC maturation and decreased actin polymerization both contribute to the Treg effect on viral infection, and they limit cell-mediated viral transfer. The amplitude of the Treg effect in our study was modest, but not very far from that reported in previous studies. Indeed, it is important to note even chemical inhibitors of cytoskeleton rearrangement never achieved full inhibition of HIV transmission. For example, high doses of latrunculin A resulted in only 25% decrease of cell-to-cell HIV transmission (41). In another study, treatment of primary CD4^+^ T cells with the glycoprotein Slit2, which also affected actin polymerization, resulted in less than 50% decrease in HIV infection (42). Similarly, low doses of Cytochalasin D decreased actin polymerization in DC:CD4 T cell co-cultures, but did not significantly decrease the number of DC:T cell aggregates, comparable to the Treg effect, resulting in a significant decrease of HIV-1 transfer to T cells by only ~25% (43). Similarly, a small but significant Treg-mediated decrease of DC maturation could significantly impact the ability of these DC to activate naïve CD4 T cells (20).

We also studied which molecular mechanisms are used by Treg to control HIV replication in DC:Tcon clusters. Our results clearly indicate an involvement of CTLA-4 in Treg activity. CTLA-4 expression by Treg and interaction with its ligands lead to decreased DC maturation (8, 24), which could explain the reduced expression levels of CD83 and CD40 in Treg-containing cultures. Another possibility is that ligation of Treg CTLA-4 induces their secretion of TGF-β, which can impair DC maturation (44). TGF-β can also inhibit expression of molecules important in IS formation such as ICAM and CD40L (35). Moreover, TGF-β could have a direct effect on HIV replication (45). However, our preliminary studies with a blocking anti-TGF-β Ab did not suggest a critical role for TGF-β in the effect of Treg (data not shown).

Our results also clearly indicate that cAMP is an additional mechanism used by Treg to control HIV infection. Increased intracellular cAMP levels block virus transfer from DC to CD4^+^ T cells (46) and prevent viral release from HIV-infected T cells (47). cAMP signaling could act at different levels. First, it could affect DC maturation, as cAMP affects expression of co-stimulatory molecules by DC and thus their immunogenicity (48). Second, increased intracellular levels of cAMP in the DC could directly decrease HIV replication and trafficking. Indeed, cAMP activates CREB, which competes with NF-κB, thus suppressing the HIV LTR transcription activity in infected cells (49). Alternatively, cAMP has been shown to induce HIV lysosomal degradation in immature DC (46). Downstream of cAMP activation, PKA directly phosphorylate monomeric actin, which causes a significant decrease in actin polymerization (50). Consistent with this hypothesis, we found that treatment of DC:Tcon co-cultures with a cAMP inducing agent (forskolin) decreased actin polymerization at the IS (Figure S2 in Supplementary Material). This mechanism could thus contribute to decreased HIV movement to the IS and possibly to its degradation in the DC.

Taken together, with previous data (14, 15), this new study shows that Treg can control *in vitro* HIV infection in all its three major targets (macrophages, activated T con, and DC), although the effect on DC was limited. Such a range of targets suggest that Treg might have a cumulative effect, as they could act on different steps of the viral life cycle. However, it remains uncertain whether Treg can decrease HIV replication *in vivo*. This role is suggested by the fact that anti-CTLA-4 treatment of SIV-infected macaques during the acute phase led to enhanced viral replication (51). Similarly, treatment of chronically infected African-green monkeys by Ontak (IL-2R-diphtheria toxin) increased viral load (52), although it is unlikely that Treg depletion was the only mechanism involved in these results. Accordingly, expansion of Treg in exposed non-infected individuals has been hypothesized to contribute to their resistance to infection (53). Similar protective function of Treg expansion has also been reported during the acute phase of SIV infection of SHIV-vaccinated macaques or African-green monkeys (54, 55). In addition to their effect on viral replication, Treg control of immune responses, including DC activation, could mediate protection of the host from tissue damages due to exacerbated immune responses to viral antigens. This effect was shown in a murine model of neuro-AIDS (14) and was reminiscent of a similar role played by Treg in other viral infections (56, 57).

## Author Contributions

Maria E. Moreno-Fernandez and Jara J. Joedicke performed experiments and analyzed the data; Maria E. Moreno-Fernandez and Claire A. Chougnet designed the experiments and wrote the manuscript.

## Conflict of Interest Statement

The authors declare that the research was conducted in the absence of any commercial or financial relationships that could be construed as a potential conflict of interest.

## Supplementary Material

The Supplementary Material for this article can be found online at http://www.frontiersin.org/Journal/10.3389/fimmu.2014.00199/abstract

Figure S1 **The effect of Treg is independent of the use of VLP**. **(A)** DCs were infected with HIV_BaL_ alone, without VLP for 3 days and cultured with CFSE-labeled Tcon. Treg were added or not to DC:Tcon cultures at a 1:10:1 (DC:Tcon:Treg) ratio and co-cultured for 24 h. After exclusion of dead cells, the percentage of HIV-p24Gag^+^ cells was measured in the gated DC:Tcon (CFSE^+^HLADR^+^). One representative experiment is shown. Percentage of HIV-p24Gag^+^ cells is indicated in each panel. INF, infected. **(B)** Summary of all experiments (*n* = 6).

Figure S2 **Cytochalasin D and Forskolin treatment decrease actin polymerization at the immunological synapse in DC:Tcon clusters**. The upper panel shows the effect of Cytochalasin D treatment on actin polymerization at the DC:Tcon IS. The lower panel shows the effect of Forskolin treatment on actin polymerization at the DC:Tcon IS.
