# Supplementary figures and images for: Regulatory T Cells Diminish HIV Infection in Dendritic Cells – Conventional CD4+ T Cell Clusters
Source: Front Immunol. 2014 May 8;5:199. doi: 10.3389/fimmu.2014.00199 (PMC4021135; doi:10.3389/fimmu.2014.00199)

## Supplementary Figure 1

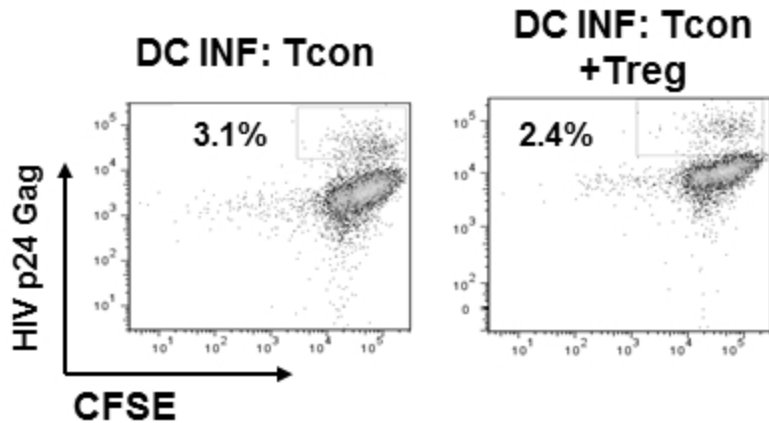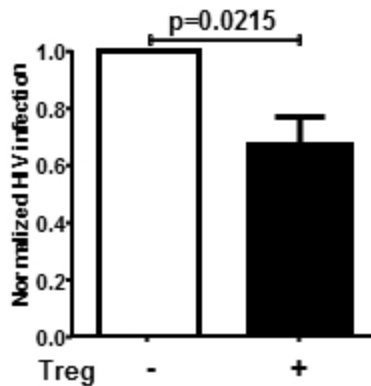

Supplement: Figure S1 — The effect of Treg is independent of the use of VLP. (A) DCs were infected with HIVBaL alone, without VLP for 3 days and cultured with CFSE-labeled Tcon. Treg were added or not to DC:Tcon cultures at a 1:10:1 (DC:Tcon:Treg) ratio and co-cultured for 24 h. After exclusion of dead cells, the percentage of HIV-p24Gag+ cells was measured in the gated DC:Tcon (CFSE+HLADR+). One representative experiment is shown. Percentage of HIV-p24Gag+ cells is indicated in each panel. INF, infected. (B) Summary of all experiments (n = 6). [file Presentation1.PDF]

## Supplementary Figure 2

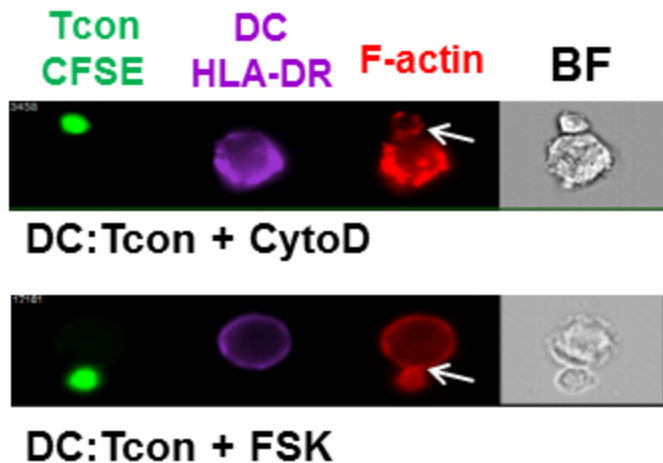

Supplement: Figure S2 — Cytochalasin D and Forskolin treatment decrease actin polymerization at the immunological synapse in DC:Tcon clusters. The upper panel shows the effect of Cytochalasin D treatment on actin polymerization at the DC:Tcon IS. The lower panel shows the effect of Forskolin treatment on actin polymerization at the DC:Tcon IS. [file Presentation2.PDF]
